# Highly effective liquid and solid phase extraction methods to concentrate radioiodine isotopes for radioiodination chemistry

**DOI:** 10.1002/jlcr.3994

**Published:** 2022-08-07

**Authors:** Christopher Davis, Chun Li, Ruirui Nie, Norman Guzzardi, Barbara Dworakowska, Pragalath Sadasivam, John Maher, Eric O. Aboagye, Zhi Lu, Ran Yan

**Affiliations:** ^1^ School of Biomedical Engineering and Imaging Sciences, St. Thomas' Hospital King's College London London UK; ^2^ Department of Nuclear Medicine First Affiliated Hospital of Dalian Medical University Dalian China; ^3^ School of Cancer and Pharmaceutical Studies, Guy's Hospital King's College London London UK; ^4^ Department of Immunology Eastbourne Hospital East Sussex UK; ^5^ Guy's Hospital Leucid Bio Ltd London UK; ^6^ Cancer Imaging Centre, Department of Surgery and Cancer Imperial College London UK

**Keywords:** iodotriazole, phase transfer reagent, radioiodination, radioiodine

## Abstract

Radioactive iodine isotopes play a pivotal role in radiopharmaceuticals. Large‐scale production of multi‐patient dose of radioiodinated nuclear medicines requires high concentration of radioiodine. We demonstrate that tetrabutylammonium chloride and methyltrioctylamonium chloride are effective phase transfer reagents to concentrate iodide‐124, iodide‐125 and iodide‐131 from the corresponding commercial water solutions. The resulting concentrated radioiodide, in the presence of either phase transfer reagent, does not hamper the chemical reactivity of aqueous radioiodide in the copper (II)‐mediated one‐pot three‐component click chemistry to produce radioiodinated iodotriazoles.

## Graphical abstract

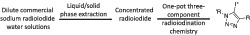

## 1 INTRODUCTION

**FIGURE 1:**
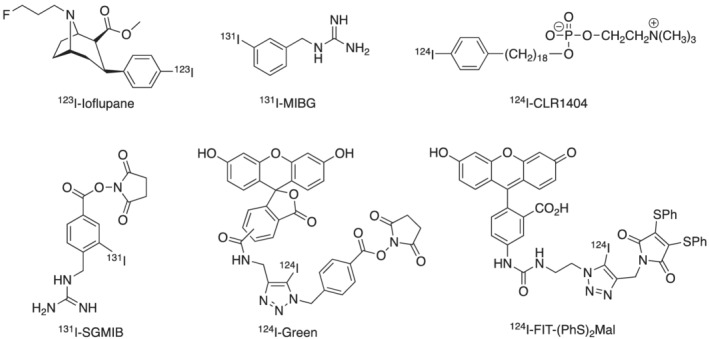
Radioiodinated nuclear medicines and bioconjugation reagents

Radioiodine isotopes play a key role in nuclear medicine.^1^ It has several radioisotopes that are routinely used in clinical practice for both nuclear imaging and radionuclide therapy. These include iodine‐123 (t_1/2_ = 13.2 h, γ) for SPECT imaging, iodine‐124 (t_1/2_ = 4.18 days, β^+^) for PET imaging, iodine‐125 (t_1/2_ = 59.4 days, γ and Auger electron) for brachytherapy and iodine‐131 (t_1/2_ = 8.2 days, β and γ) for SPECT imaging and radionuclide therapy.^1^ A key advantage of radioiodine is that the same bioactive compound can be labelled with any radioiodine isotope using the same chemistry.^2^ Thus, theranostic radiopharmaceuticals with identical in vivo pharmacokinetics can often be developed. Figure 1 summarised a few representative radioiodine‐based radiopharmaceuticals and bioconjugation reagents. Illustrating this, ^123^I‐Ioflupane, a well‐established DAT‐scanning agent enables the imaging of Parkinson's disease using SPECT.^3^ Moreover, ^131^I‐MIBG is used for the treatment of neuroendocrine tumours,^4^ while [^124^I]CLR1404 PET/CT has successfully detected high‐grade primary and metastatic brain tumours in a recent clinical trial.^5^ ^131^I‐SGMIB is a deiodination resistant prosthetic group for labelling of peptides and proteins.^6^ We have developed two iodine‐124 based novel dual PET and fluorescent bioconjugation reagents, ^124^I‐Green for antibody labelling^7^ and ^124^I‐FIT‐(PhS)_2_Mal for cell tracking,^8^ respectively.

Both ^124^I‐Green and ^124^I‐FIT‐(PhS)_2_Mal were prepared by a one‐pot three‐component radioiodination chemistry. This copper (II)‐mediated radiochemistry involves the reaction of an alkyne and an azide in organic phase, and sodium radioiodide in water to form an iodiotriazole.^9^, ^10^, ^11^ This approach has been frequently adopted by other researchers to prepare new radioiodinated reagents for nuclear imaging and radionuclide therapy.^12^, ^13^, ^14^ Despite its versatility, this heterogenous radiochemical reaction can only tolerate very small amount of water (<10 μl) within which the radioiodide is carried. All four major radioiodine isotopes are supplied as sodium radioiodide in NaOH or Na_2_S_2_O_3_ water solution. Commonly, the concentrations of the commercial sodium radioiodide range from 0.1 to 11 MBq/μl depending on the regional supplier. So far, there is no effective method to concentrate radioiodine. Thus, it is very challenging to employ this one‐pot three‐component radioiodination chemistry to prepare multi‐patient dose of radioiodinated radiopharmaceuticals for clinical applications.

Herein, we report simple liquid and solid phase extraction methods to concentrate radioiodine for scaling up the one‐pot three‐component radioiodination chemistry. The radioiodine can be efficiently extracted from the water solution by either dichloromethane (DCM) extraction using the phase transfer reagent, tetrabutylammonium chloride or by passing through a tC18 cartridge in the presence of the phase transfer reagent, methyltrioctylammonium chloride. The concentrated radioiodine with either phase transfer reagent can be readily used for the one‐pot three‐component radioiodination reactions to provide radiochemical yields (RCYs) comparable to the same reactions using sodium radioiodide in water.

## 2 MATERIAL AND METHODS

### 2.1 General information

^1^H and ^13^C NMR spectra were recorded at room temperature on a Bruker Avance 400 instrument operating at the frequency of 400 MHz for ^1^H and 100 MHz for ^13^C. Chemical shifts are reported in ppm relative to chloroform (δ 7.26, s) or dimethyl sulfoxide (δ 2.48, m), and coupling constants (J) are given in Hertz. High‐resolution mass data were recorded on a Waters Acquity UPLC‐ Xevo G2‐XS QToF. HPLC analysis was performed with an Agilent 1200 HPLC system equipped with a 1200 series diode array detector. Radio‐HPLC analysis was performed with an Agilent 1200 HPLC system equipped with a series diode array detector and Raytest GABI Star radioactivity detector. Radioactivity was measured by an ionisation chamber (Capintec). All reagents were purchased from Sigma‐Aldrich and were used without further purification. Reductant free [^124^I]NaI was purchased from Advanced Center Oncology Macerata (ACOM) in 0.02 M NaOH (pH 12.4) aqueous solution; radioactivity concentration: 0.20 MBq/μl; and radionuclide purity >99.2%. Reductant free [^125^I]NaI was purchased from Hartmann Analytic (product number I‐RB‐31) in 0.04 M NaOH (pH > 12) aqueous solution; radioactivity concentration: 3.70 MBq/μl; and radionuclide purity >99.1%. Clinical grade [^131^I]NaI was purchased from HTA Co., Ltd., China, in sodium thiosulphate (pH 7‐9) aqueous solution; radioactivity concentration: 0.18 MBq/μl; and radionuclide purity >99.9%. Sep‐Pak C18 Plus Light Cartridge (130 mg Sorbent per Cartridge, 55–105 μm) and Sep‐Pak tC18 Plus Light Cartridge (145 mg Sorbent per Cartridge, 37–55 μm) were purchased from Waters.

### 2.2 Concentration of radioiodine with liquid phase extraction

Tetrabutylammonium chloride, methyltrioctylammonium chloride or benzyltriethylammonium chloride (1.0 mg) in water (50 μl) was added to aqueous radioiodide (10–370 MBq, 500 μl). The solution was shaken gently for 1 min. Ethyl acetate or DCM (500 μl) was then added to this solution and shaken vigorously for another 5 min. The mixture was left to settle for 10 min till the two liquid layers were separated. The aqueous layer was carefully removed with a pipette. The radioactivity in the organic layer and the aqueous layer was measured with a Capintec ionisation chamber. DCM was evaporated under a stream of N_2_, and the dried residual that contains most of radioiodine was used for radiolabelling.

### 2.3 Concentration of radioiodine with solid phase extraction

Tetrabutylammonium chloride, methyltrioctylammonium chloride or benzyltriethylammonium chloride (1.0 mg) in water (50 μl) was added to aqueous radioiodide (10‐370 MBq, 500 μl). The solution was shaken gently for 1 min and passed through either a C18 light or tC18 cartridge. The cartridge was washed with water (1.0 ml) and dried with N_2_. The radioactivity on the cartridge was released with acetonitrile (1.0 ml). The radioactivity in the organic phase, the aqueous phase and cartridge was measured with a Capintec ionisation chamber. Acetonitrile was evaporated under a stream of N_2_, and the dried residual that contains most of radioiodine was used for radiolabelling.

### 2.4 Radiochemistry

^124/125/131^I‐FIT‐(PhS)_2_Mal:

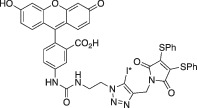




Copper (II) chloride (3.4 mg, 25.3 μmol), triethylamine (4.7 μl, 34.2 μmol, 1.38 equiv.) and bathophenanthroline (850 μg, 2.5 μmol, 10% mol) were mixed in anhydrous acetonitrile (500 μl). The resulting suspension (31.2 μl) was added to *N*‐propargyl‐3,4‐dithiophenolmaleimide (1.6 μmol) in anhydrous DMF (31.2 μl). The resulting red suspension (20 μl) was added to a mixture of 5 [3‐(2‐azidoethyl)ureido]‐fluorescein (0.5 μmol) in acetonitrile (10 μl) and either the iodine‐124 (~60 MBq) concentrated with methyltrioctylammonium chloride (1.0 mg) in water (3.0 μl) or the iodine‐125 (~50 MBq) concentrated with tetrabutylammonium chloride (1.0 mg) in water (3.0 μl), or the iodine‐131 (~370 MBq) concentrated with tetrabutylammonium chloride (1.0 mg) in water (3.0 μl). The reaction was heated at 60°C for 1.5 h before quenching with DMSO (100 μl), followed by water/MeOH (4:1, 1.0 ml). The resulting solution was purified by HPLC using a ZORBAX column (300SB‐C18, 9.4 × 250 mm, 5 μm) with the following eluent: water (0.1% TFA) as solvent A and methanol (0.1% TFA) as solvent B, went from 70% B to 75% B in 15 min, increased to 90% B in 5 min and went back to 70% B in 5 min with a flow rate of 2.5 ml/min. The retention time of the title compounds was 11.55 min.

1‐Benzyl‐5‐[^125^I]iodo‐4‐(3‐phenylpropyl)‐1H‐1,2,3‐triazole:

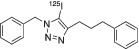




Copper (II) chloride (134 μg, 1.0 μmol), triethylamine (151 μg, 1.5 μmol, 1.5 equiv.) and bathophenanthroline (33 μg, 0.1 μmol, 0.1 equiv.) were mixed in anhydrous acetonitrile (20 μl) and sonicated for 2 min. 5‐Phenyl‐1‐pentyne (144 μg, 1.0 μmol) was dissolved in anhydrous acetonitrile (20 μl) containing TEA·HCl (80 μg, 0.58 μmol) and then added to the above mixture. The resulting mixture was added to either the Na^125^I (~4 MBq) in aqueous NaOH (6 μl, 0.04 M) or iodine‐125 (~4 MBq) concentrated with methyltrioctylammonium chloride (1.0 mg) in water (6 μl). Benzyl azide in a DMF:DCM 1:1 solution (3.0 μmol, 20 μl, 3.0 equiv.) was added to the reaction and was heated in a heating block at 60°C for 1.5 h. The reaction mixture was quenched with 20% MeOH in water (1.0 ml). The resulting solution was purified by HPLC using a Chromolith® SemiPrep RP‐18 endcapped 100‐10 monolithic HPLC‐column with the following eluent: water (0.1%, formic acid) as solvent A and methanol (0.1%, formic acid) as solvent B, went from 5% B to 95% B in 30 min and went back to 5% B in 5 min with a flow rate of 3 ml/min. The retention time of the title compounds was 22.20 min.

4‐(2‐Fluoro‐ethyl)‐5‐[^125^I]iodo‐1‐phenyl‐1H‐[1,2,3]triazole:




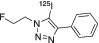




Copper (II) chloride (134 μg, 1.0 μmol), triethylamine (151 μg, 1.5 μmol, 1.5 equiv.) and bathophenanthroline (33 μg, 0.1 μmol, 0.1 equiv.) were mixed in anhydrous acetonitrile (20 μl) and sonicated for 2 min. Phenylacetylyne (102 μg, 1.0 μmol) was dissolved in anhydrous acetonitrile (20 μl) containing TEA·HCl (80 μg, 0.58 μmol) and then added to the above mixture. The resulting mixture was added to the either Na^125^I (~4 MBq) in aqueous NaOH (6 μl, 0.04 M) or iodine‐125 (~4 MBq) concentrated with methyltrioctylammonium chloride (1.0 mg) in water (6 μl) in a screw cap Wheaton vial. Subsequently, the solution of 1‐azido‐2‐fluoroethane in DMF (2.0 μmol, 20 μl, 2.0 equiv.) was added to the reaction and was kept at room temperature for 1.5 h. The reaction mixture was quenched with 20% MeOH in water (1.0 ml). The resulting solution was injected to the HPLC using a Chromolith® SemiPrep RP‐18 endcapped 100‐10 monolithic HPLC‐column with the following eluent: water (0.1% TFA) as solvent A and methanol (0.1% TFA) as solvent B, went from 40% B to 95% B in 10 min and went back to 40% B in 5 min with a flow rate of 5 ml/min. The retention time of the title compound was 4.17 min.

### 2.5 Synthetic chemistry

1‐Benzyl‐5‐iodo‐4‐(3‐phenylpropyl)‐1H‐1,2,3‐triazole:




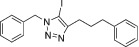




Copper(I) iodide (267 mg, 1.4 mmol), triethylamine (200 μl, 1.4 mmol) and bathophenanthroline (47 mg, 0.14 mmol) were mixed in anhydrous DMF (14 ml). Next, 5‐phenyl‐1‐pentyne (200 mg, 1.4 mmol) was added to the above mixture and stirred at RT for 5 min. Next, N‐iodosuccinimide (470 mg, 2.1 mmol) was introduced to the system and stirred at RT for another 5 min. Finally, benzyl azide (2.8 ml, 1.4 mmol, 0.5 M in dichloromethane) was added to the reaction mixture and stirred at RT overnight under N_2_ atmosphere. The reaction was quenched by MeOH and filtered through Celite. The filtrate was concentrated in vacuo, and residual was partitioned between EtOAc/H_2_O. The combined organic layers were dried over MgSO_4_, concentrated in vacuo and purified with flash chromatography (EtOAc/n‐Hexane and then MeOH/DCM) to yield title compound as colourless oil (280 mg, 50%).

^1^H NMR (400 MHz, CDCl_3_) δ 7.31–7.08 (10H, m, Ph), 5.49 (2H, s, PhCH_2_N), 2.68–2.61 (4H, m, CH_2_CH_2_CH_2_), 2.05–1.97 (2H, m, CH_2_CH_2_CH_2_). ^13^C NMR (100 MHz, CDCl_3_) δ 152.06, 141.84, 134.59, 128.92, 128.58, 128.45, 128.41, 127.78, 125.91, 78.71, 54.16, 35.36, 30.48, 25.77. HRMS (EI, ^m^/_z_) [M + H]^+^: calc. for C_18_H_19_IN_3_ 404.0624; found: 404.0511.

4‐(2‐Fluoro‐ethyl)‐5‐iodo‐1‐phenyl‐1H‐[1,2,3]triazole:




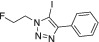




Copper(I) iodide (48 mg, 0.25 mmol) and triethylamine (35 μl, 0.25 mmol) were dissolved in anhydrous DMF (0.1 ml). *N*‐iodosuccinimide (62 mg, 0.28 mmol, 1.1 equiv.), phenylacetylene (26 mg, 0.25 mmol) and 1‐azido‐2‐fluoroethane (0.25 mmol) in anhydrous DMF (1 ml) were added and the mixture was stirred overnight at room temperature under nitrogen. The reaction was quenched with water (10 ml), and the product mixture was extracted with DCM (3 × 15 ml). The organic layer was washed with brine (30 ml) and dried over MgSO_4_. The solvents were removed in vacuo. The crude product was purified by flash column chromatography on silica eluting with 20–50% EtOAc in petroleum ether to yield title compound as colourless oil (14 mg, 18%). ^1^H NMR (400 MHz, d_6_‐DMSO) δ 7.89–7.88 (2H, m, Ph), 7.54–7.50 (2H, m, Ph), 7.45–7.43 (1H, m, Ph), 4.97 (2H, dt, J_F,H_ = 48 Hz, J_1,2_ = 4 Hz, CH_2_F), 4.84 (2H, dt, J_F,H_ = 28 Hz, J_1,2_ = 4 Hz, CH_2_CH_2_F). ^13^C NMR (100 MHz, d_6_‐DMSO) δ 149.18, 131.00, 129.15, 128.85, 127.54, 83.02, 81.33, 51.24; HRMS (EI, ^m^/_z_) [M + H]^+^: calc. for C_10_H_10_FIN_3_ 317.9903; found: 318.0618.

## 3 RESULTS AND DISCUSSION

### 3.1 Extraction of radioiodide from aqueous solution

Three commercially available phase transfer reagents, tetrabutylammonium chloride, methyltrioctylammonium chloride or benzyltriethylammonium chloride (1.0 mg), were each dissolved in Na^125^I (10 MBq) water solution (500 μl) containing NaOH (0.04 M). The resulting solution was extracted with either ethyl acetate or DCM, respectively. The extraction efficiency is summarised in Table 1. Ethyl acetate proved ineffective to extract iodide‐125 from water with all three phase transfer reagents. In contrast, over 90% of iodide‐125 was extracted to the DCM phase when tetrabutylammonium chloride or methyltrioctylammonium chloride was used as the phase transfer reagent, while only 16% of iodide‐125 was transferred to DCM phase by benzyltriethylammonium chloride. This lower efficiency could be because of the lower lipophilicity of benzyltriethylammonium chloride (log p 0.07), when compared to tetrabutylammonium chloride (log p 2.01) or methyltrioctylammonium chloride (log p 5.52).^15^ The DCM was removed under nitrogen and the resulting organic iodide‐125 could then be used for radiolabelling. Next, we investigated the use of a solid phase extraction method to concentrate Na^125^I (10 MBq) from the water solution of NaOH (500 μl, 0.04 M). The iodide‐125 aqueous solutions containing tetrabutylammonium chloride, methyltrioctylammonium chloride or benzyltriethylammonium chloride (1.0 mg), respectively, were passed through either a Sep‐Pak C18‐light or a tC18‐light cartridge. After washing the cartridge with water (1.0 ml), the radioactivity was then released with acetonitrile. Around 88% of iodide‐125 was transferred to the organic phase by methyltrioctylammonium chloride using a tC18 light cartridge. A lower iodide‐125 solid phase extraction efficiency that ranged from 11–71% was observed when tetrabutylammonium chloride or benzyltriethylammonium chloride was used as the phase transfer reagents. Once again, we believe that the higher lipophilicity of the methyltrioctylammonium chloride and tC18 light cartridge play a key role in the higher efficiency of iodide‐125 extraction using this method. The acetonitrile was removed under nitrogen, and the resulting dried organic iodide‐125 could be used for radiolabelling.

**TABLE 1:** Comparison of phase transfer reagents for radioiodine extraction

Entry	Phase transfer reagents	Iodine‐125 extraction efficiency				Iodine‐131 extraction efficiency	Iodine‐124 extraction efficiency
		Ethyl acetate	DCM	C18‐light cartridge	tC18‐light cartridge	DCM	tC18‐light cartridge
1	tetrabutyl ammonium chloride	15%	94 ± 3% (*n* = 7)	26%	71%	92 ± 2% (*n* = 6)	62%
2	methyltrioctyl ammonium chloride	20%	90%	74%	88 ± 5% (*n* = 9)	N/T	89 ± 5% (*n* = 3)
3	benzyltriethyl ammonium chloride	0%	16%	11%	N/T	N/T	N/T

Abbreviation: N/T, not tested.

The concentrations of the commercial clinical grade Na^131^I water solution and reductant free Na^124^I in water solution were 0.18 and 0.20 MBq/μ, respectively. These concentrations were too low to be employed to prepare sufficient radioiodinated compounds for nuclear imaging or radiotherapy applications using the one‐pot three‐component radioiodination chemistry because the reaction can only tolerate <10 μl of water. Therefore, we decided to apply the liquid phase and the solid phase radioiodine extraction methods to concentrate both radioiodide. When tetrabutylammonium chloride was used as phase transfer reagent, ~92% of iodide‐131 was extracted by DCM from the clinical grade [^131^I]NaI/Na_2_S_2_O_3_ aqueous solution. On the other hand, ~89% of iodide‐124 was recovered using methyltrioctylammonium chloride and a tC18 light cartridge. Both methods enable us to concentrate several hundred MBq of iodide‐131 and iodide‐124. In addition, the solid phase extraction method is suitable for concentrating radioiodine using an automated synthesiser such as the GE FASTlab or the Eckert & Ziegler system.

**SCHEME 1:**
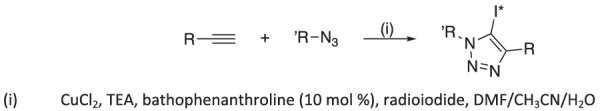
One‐pot three‐component radioiodination

To test the reactivity of the concentrated radioiodine using either tetrabutylammonium chloride or methyltrioctylammonium chloride, we conducted several copper (II) mediated one‐pot three‐component radioiodination click reactions (Scheme 1), and the corresponding radiochemical yields (RCYs) were summarised in Table 2. Initially, we used the dried iodide‐125/tetrabutylammonium chloride without water for the radioiodination between the *N*‐propargyl‐3,4‐dithiophenolmaleimide and 5‐[3‐(2‐azidoethyl)ureido]‐fluorescein. Poor RCYs of <20% were observed. However, when water (3‐6 μl) was added to the reaction mixture, excellent RCYs of 75%, 87% and 76% were observed for ^125^I‐FIT‐(PhS)_2_Mal, ^131^I‐FIT‐(PhS)_2_Mal and ^124^I‐FIT‐(PhS)_2_Mal, respectively (Table 2, Entry 1). These radiochemical yields are comparable to those previously reported for the cell labelling reagent, ^124^I‐FIT‐(PhS)_2_Mal (~71%) that was prepared using Na^124^I in water as the source of radioiodide. Next, we compared the RCYs of the one‐pot three‐component formation of the 1‐benzyl‐5‐[^125^I]iodo‐4‐(3‐phenylpropyl)‐1H‐1,2,3‐triazole using either the dried iodide‐125/methyltrioctylammonium chloride or Na^125^I in water. Comparable RCYs of 55% and 61%, respectively, were observed (Table 2, Entry 2). In addition, similar RCYs of 4‐(2‐fluoro‐ethyl)‐5‐[^125^I]iodo‐1‐phenyl‐1H‐[1,2,3]triazole were also obtained using either the dried iodide‐125/methyltrioctylammonium chloride or Na^125^I in water (Table 2, Entry 3). All three examples indicate that the concentrated radioiodide using either tetrabutylammonium chloride or methyltrioctylammonium chloride retains the chemical reactivity in the one‐pot three‐component radioiodination chemistry. The identity of all three radioiodinated compounds was confirmed by co‐eluting with their non‐radioactive reference compounds (Figure S1–S3). It is worth noting that other radioiodination methodologies such as silver^16^ or palladium^17^ mediated radioiodination chemistry could also benefit from the above radioiodine concentration methods to produce multiple patient doses of radioiodinated nuclear medicines.

**TABLE 2:** One‐pot three‐component radioiodination reactions

Entry	Alkynes	Azides	Iodinated triazoles	RCYs (%)
1	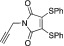	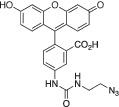	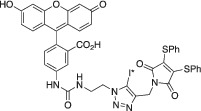	75 ± 3^a^
				87 ± 5^b^
				76 ± 5^c^
				71 ± 1^d^
2	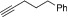	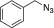	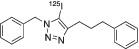	55 ± 5^e^
				61 ± 3^f^
3	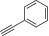	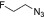	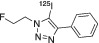	50 ± 4^e^
				54 ± 9^f^

^a^ I* = ^125^I, tetrabutylammonium chloride was used, *n* = 3.

^b^ I* = ^131^I, tetrabutylammonium chloride was used, *n* = 5.

^c^ I* = ^124^I, methyltrioctylammonium chloride was used, *n* = 3.

^d^ I* = ^124^I, literature data^8^ no phase transfer reagent was used.

^e^ Methyltrioctylammonium chloride was used, *n* = 3–4.

^f^ No phase transfer reagent was used, *n* = 3–4.

## 4 CONCLUSION

We have developed highly efficient liquid and solid phase extraction methods for concentrating radioactive iodine using tetrabutylammonium chloride and methyltrioctylammonium chloride as the phase transfer reagents, respectively. The reactivity of the concentrated radioactive iodide, in the presence of either tetrabutylammonium chloride or methyltrioctylammonium chloride, does not hamper the RCYs of the copper (II) mediated one‐pot three‐component radioiodination click reaction. The cartridge‐based solid phase extraction method using methyltrioctylammonium chloride can be readily implemented on an automated synthesiser to produce the radioiodinated triazoles using this copper (II) mediated reaction of azides, alkynes and radioiodide.

## CONFLICT OF INTEREST

The authors report no conflict of interest in this work.

## Supporting information




**Figure S1.** HPLC chromatogram of the coelution of ^125^I‐FIT‐(PhS)_2_Mal with its non‐radioactive reference compound
**Figure S2.** HPLC chromatogram of the coelution of 1‐benzyl‐5‐[^125^I]iodo‐4‐(3‐phenylpropyl)‐1H‐1,2,3‐triazole with its non‐radioactive reference compound
**Figure S3.** HPLC chromatogram of the coelution of 4‐(2‐fluoro‐ethyl)‐5‐[^125^I]iodo‐1‐phenyl‐1H‐[1,2,3]triazole with its non‐radioactive reference compound
^**1**^ **H and** ^**13**^ **C NMR spectra**

## Data Availability

The data that support the findings of this study are available in the supporting information of this article. For the purpose of open access, the author has applied a Creative Commons Attribution (CC BY) licence (where permitted by UKRI, ‘Open Government Licence’ or ‘Creative Commons Attribution No‐derivatives (CC BY‐ND) licence’ may be stated instead) to any Author Accepted Manuscript version arising.
