# Supplementary material for: Highly effective liquid and solid phase extraction methods to concentrate radioiodine isotopes for radioiodination chemistry
Source: J Labelled Comp Radiopharm. 2022 Aug 7;65(10-11):280–7. doi: 10.1002/jlcr.3994 (PMC9773003; doi:10.1002/jlcr.3994)

**Supporting Information**

**Highly Effective Liquid and Solid Phase Extraction Methods to Concentrate Radioiodine Isotopes for Radioiodination Chemistry**

Christopher Davis^1^, Chun Li^2^, Ruirui Nie,^2^ Norman Guzzardi^1^, Barbara Dworakowska^1^, Pragalath Sadasivam^1^, John Maher^3,4,5^, Eric O. Aboagye^6^, Zhi Lu^2^, and Ran Yan^1^

^1^King’s College London, School of Biomedical Engineering and Imaging Sciences, St. Thomas’ Hospital, SE1 7EH, London, United Kingdom;

^2^Department of Nuclear Medicine, First Affiliated Hospital of Dalian Medical University, People’s Republic of China;

^3^King’s College London, School of Cancer and Pharmaceutical Studies, Guy’s Hospital, Third floor Bermondsey Wing, Great Maze Pond, London SE1 9RT, United Kingdom;

^4^Department of Immunology, Eastbourne Hospital, Kings Drive, Eastbourne, East Sussex, BN21 2UD, United Kingdom;

^5^Leucid Bio Ltd., Guy’s Hospital, Great Maze Pond, London SE1 9RT, UK;

^6^Cancer Imaging Centre, Department of Surgery & Cancer, Imperial College, London SW7 2AZ, United Kingdom.

**HPLC chromatograms**

**Figure S1.** Coelution of ^125^I-FIT-(PhS)_2_Mal with its nonradioactive reference compound. HPLC conditions: Chromolith column (300SB-C18, 9.4 X 250 mm, 5 µm) with the following eluent: water (0.1% TFA) as solvent A and methanol (0.1% TFA) as solvent B, kept at 65% of B for 3 min, went from 65% B to 75% B in 12 min, went from 75% B to 100% B in 2 min, kept at 100% of B for 1 min, and went back to 65% B in 1 min with a flow rate of 5 mL/min.

**Figure S2.** Coelution of 1-benzyl-5-[^125^I]iodo-4-(3-phenylpropyl)-1H-1,2,3-triazole with its nonradioactive reference compound. HPLC conditions: Chromolith column (300SB-C18, 9.4 X 250 mm, 5 µm) with the following eluent: water (0.1%, formic acid) as solvent A and methanol (0.1%, formic acid) as solvent B, went from 5% B to 95% B in 30 min, and went back to 5% B in 5 min with a flow rate of 3 mL/min.

**Figure S3.** Coelution of 4-(2-fluoro-ethyl)-5-[^125^I]iodo-1-phenyl-1H-[1,2,3]triazole with its nonradioactive reference compound. HPLC conditions: Chromolith column (300SB-C18, 9.4 X 250 mm, 5 µm) with the following eluent: water (0.1% TFA) as solvent A and methanol (0.1% TFA) as solvent B, went from 40% B to 95% B in 10 min and went back to 40% B in 5 min with a flow rate of 5 mL/min.

**NMR spectra**

1-Benzyl-5-iodo-4-(3-phenylpropyl)-1H-1,2,3-triazole

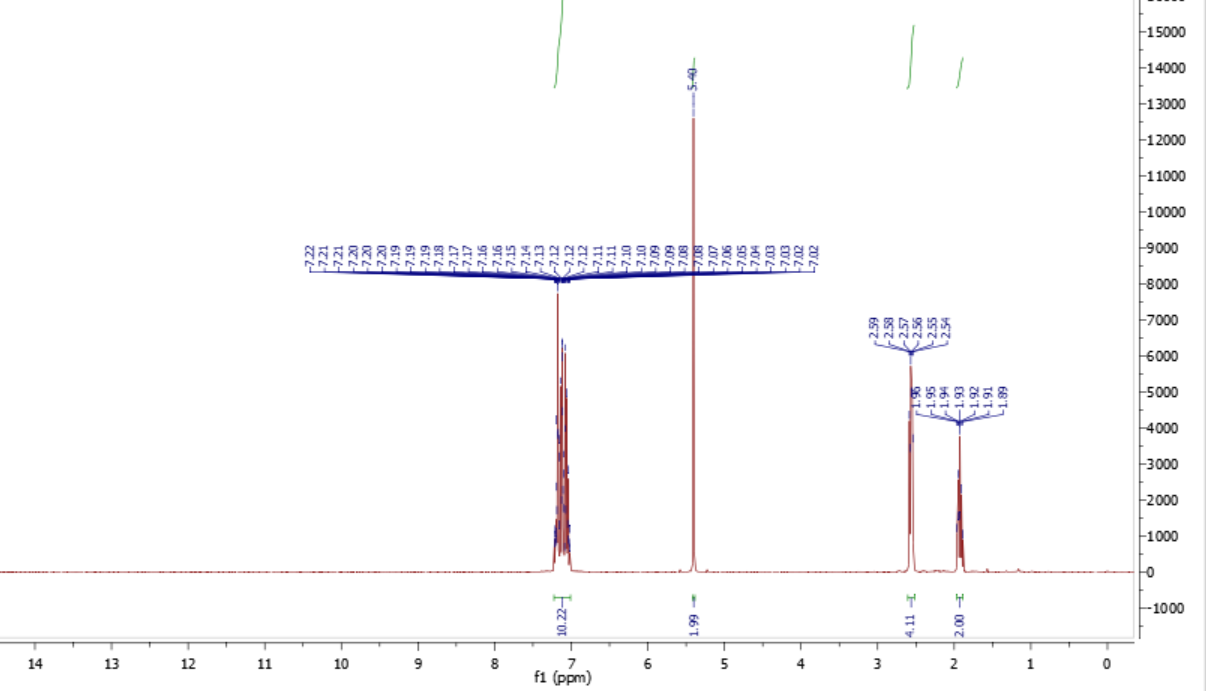


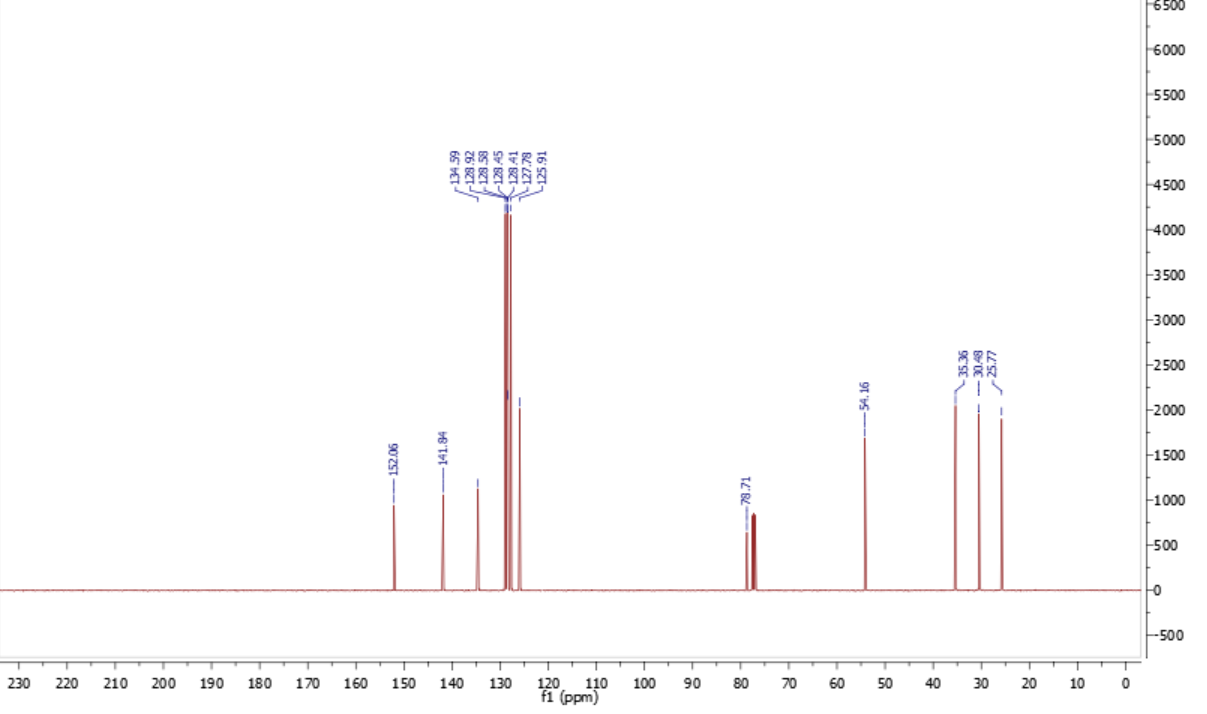


4-(2-Fluoro-ethyl)-5-iodo-1-phenyl-1H-[1,2,3]triazole

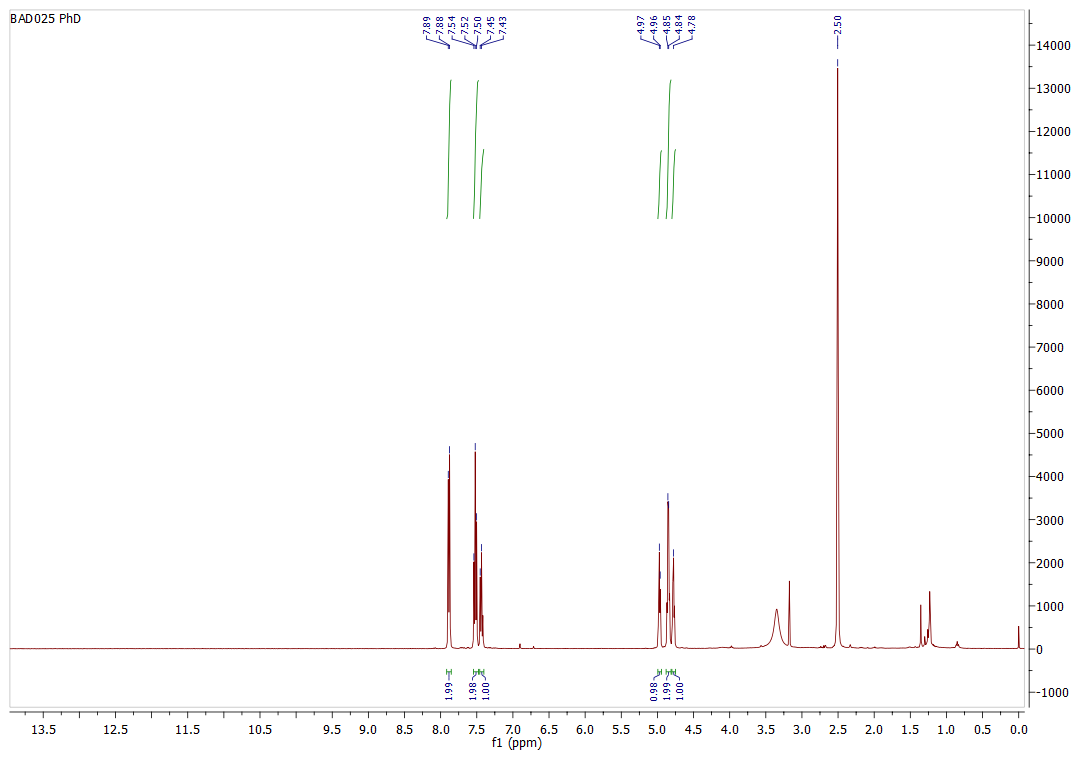


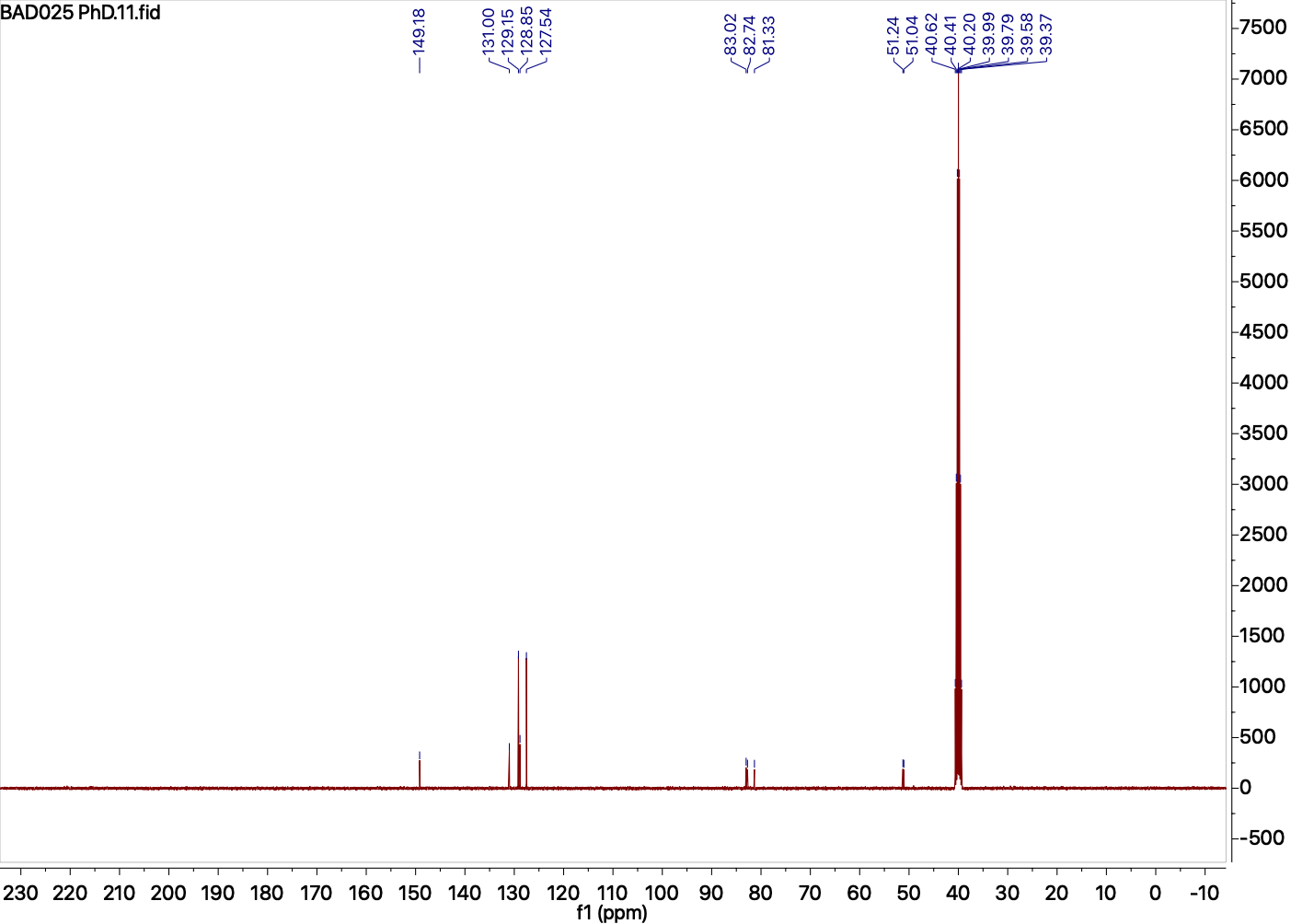

Supplement: Supplementary file 1 — Figure S1. HPLC chromatogram of the coelution of 125I‐FIT‐(PhS)2Mal with its non‐radioactive reference compound Figure S2. HPLC chromatogram of the coelution of 1‐benzyl‐5‐[125I]iodo‐4‐(3‐phenylpropyl)‐1H‐1,2,3‐triazole with its non‐radioactive reference compound Figure S3. HPLC chromatogram of the coelution of 4‐(2‐fluoro‐ethyl)‐5‐[125I]iodo‐1‐phenyl‐1H‐[1,2,3]triazole with its non‐radioactive reference compound 1 H and 13 C NMR spectra [file JLCR-65-280-s001.docx]
